# Evaluating the Efficacy of Gelatin-Chitosan-Tetraethyl Orthosilicate Calcium Hydroxide Composite as a Dental Pulp Medicament on  COX-2, PGP 9.5, TNF-α Expression and Neutrophil number

**DOI:** 10.12688/f1000research.156336.2

**Published:** 2025-01-30

**Authors:** Al-qatta Ghadah Abdulrahman, Endytiastuti Endytiastuti, Retno Ardhani, Iwa Sutardjo Rus Sudarso, Bidhari Pidhatika, Mh Busra Fauzi, Heni Susilowati, Yulita Kristanti, Juni Handajani

**Affiliations:** 1 Student of Magister Dental Science Study Program, Faculty of Dentistry, Universitas Gadjah Mada, Yogyakarta, Special Region of Yogyakarta, Indonesia; 2 Student of Magister Clinical Dental Science Study Program, Faculty of Dentistry, Universitas Gadjah Mada, Yogyakarta, Special Region of Yogyakarta, Indonesia; 3 Department of Dental Biomedical Sciences, Faculty of Dentistry, Universitas Gadjah Mada, Yogyakarta, Special Region of Yogyakarta, Indonesia; 4 Magister of Clinical Dental Science Study Program, Faculty of Dentistry, Universitas Gadjah Mada, Yogyakarta, Special Region of Yogyakarta, Indonesia; 5 Department of Pediatric Dentistry, Faculty of Dentistry, Universitas Gadjah Mada, Yogyakarta, Special Region of Yogyakarta, Indonesia; 6 Research Center for Polymer Technology, National Research and Innovation Agency of the Republic of Indonesia, Jakarta, Indonesia; 7 Center of Tissue Engineering and Regenerative Medicine, Faculty of Medicine,, Universiti Kebangsaan Malaysia, Kuala Lumpur, 55281, Malaysia; 8 Magister of Dental Science Study Program, Faculty of Dentistry,, Universitas Gadjah Mada, Yogyakarta, Special Region of Yogyakarta, Indonesia; 9 Department of Oral Biology, Faculty of Dentistry, Universitas Gadjah Mada, Yogyakarta, Special Region of Yogyakarta, Indonesia; 10 Department of Conservative Dentistry, Faculty of Dentistry, Universitas Gadjah Mada, Yogyakarta, Special Region of Yogyakarta, 55281, Indonesia

**Keywords:** Calcium Hydroxide (Ca(OH)2), Gelatin-Chitosan-Tetraethyl Orthosilicate-Calcium Hydroxide (G-CH-TEOS-Ca(OH)2) Composite, capping material, COX-2, PGP 9.5, TNF-α, neutrophils

## Abstract

**Introduction:**

Calcium hydroxide (Ca(OH) _2_) is the material of choice for pulp therapy. However, Ca(OH) _2_ has drawbacks such as toxicity, poor sealing, and tunnel defect formation. Alternative materials have been developed to provide more biocompatible materials with better dentin formation ability. The objective of this study was to evaluate the effect of composites containing gelatin (G), chitosan (CH), tetraethyl orthosilicate (TEOS), and Ca(OH) _2_, namely G-CH-TEOS-Ca (OH) _2_ (Extended data) on inflammation of the dental pulp (expression of COX-2, PGP 9.5, TNF-α, and neutrophil number).

**Materials and methods:**

A total of 16 Wistar rat models of acute pulp injury were prepared and divided into two groups, treatment and control, 8 with each. In the treatment group, we applied a pulp-capping material using G-CH-TEOS-Ca (OH) _2_ and Ca(OH) _2_. On the 1 ^st^ and 3 ^rd^ days, rats were sacrificed. Tissue samples from 4 rats in each group were processed for histological preparation. COX-2, PGP 9.5, and TNF-α were observed using immunohistochemical (IHC) staining, and neutrophil numbers were observed using hematoxylin-eosin staining. Image analysis of COX-2, PGP 9.5, and TNF-α expression was performed using ImageJ software.

**Results:**

The results showed a decrease in COX-2 expression, but not significantly while PGP 9.5 and TNF-α expression were significantly higher than those in the control group. Neutrophil numbers were lower in the treatment group than in the control group, but the difference was not statistically significant.

**Conclusion:**

The G-CH-TEOS-Ca (OH) _2_ composite material may have potential as an exposed pulp medicament by reducing inflammation (COX-2 expression and number of neutrophils) and increasing the regeneration factor (TNF-α expression) and nerve (PGP 9.5 expression).

## Introduction

Dental caries is one of the most common chronic illnesses worldwide. Tooth decay is a complex illness involving tooth structure, oral bacteria, and dietary carbohydrates. It dissolves the mineral content of the teeth and is mostly dependent on several important contributing factors. ^1^ Small surface roughness or subsurface demineralization is the first symptom, followed by cavitation, pulp involvement, swelling, abscesses, and systemic symptoms. ^2^

Severe toothache can be incapacitating, and infection and sepsis resulting from caries spreading to the dental pulp can sometimes cause major systemic consequences (e.g., spreading local infection and, very rarely, treatment-related death due to anesthesia-related complications) in addition to tooth loss. ^3^ An inflammatory reaction in the pulp is called pulpitis. ^4^ occurs primarily due to cariogenic causes and less frequently as a result of trauma, dental abnormalities, and restorative procedures. Pulp hyperemia occurs when inciting conditions are present. Pulp diseases, including acute pulpitis, chronic pulpitis, pulp degeneration, and pulp necrosis, are dental disorders that originate in pulp tissue. Dental caries that are not treated appropriately may later evolve into pulp disease. ^5^

Neutrophils are the most abundant white blood cell in humans. Neutrophils are immunological cells with unique biological characteristics that exhibit strong antibacterial activity. These cells are highly effective in phagocytosing prokaryotic and eukaryotic species, leading to their death. Their role as first responders to acute inflammation, aiding in the healing of injured tissues and removal of pathogens, is well established. Crucially, the antimicrobial activity of neutrophils is not limited to their capacity to combat bacteria within a restricted intracellular space. Neutrophils also release effectors into the extracellular space where their killing apparatus can survive and continue to operate long after death. ^6^^,^^7^

An important evolutionary enzyme that is involved in a variety of physiological and pathological processes is COX-2. It controls the expression levels of numerous serum proteins and plays a crucial role in viral infection. This enzyme significantly affects proinflammatory cytokines, and its inhibition or lack by itself does not impair the immune system’s ability to fight illness. ^8^

Protein gene product 9.5 (PGP 9.5) is a neuronal protein with a specific biological purpose. It is expressed in axons and is used to map individuals with neurodegenerative diseases. It functions as a carboxyl-terminal hydrolase and ligase. Significantly, while PGP 9.5, a highly neuron-specific protein that is frequently employed as a generic neuronal marker, has also been utilized to quantify the neural protein recovered from pulpal tissue ^7^ and to detect nerve fibers in the dental pulp. It has been shown to be helpful in identifying the A-delta and C fibers of dental pulp. ^9^ It has been hypothesized that processes leading to nerve degeneration are connected to the early loss or lack of expression of this protein. The nerve structures of the dental pulp were assessed using PGP 9.5. A potential diagnostic marker for identifying nerve fibers in inflamed tooth pulp is PGP 9.5 expression. ^10^

In response to bacterial endotoxins, such as lipopolysaccharide (LPS), macrophages preferentially release tumor necrosis factor α (TNF-α). ^9^ Numerous lines of evidence suggest that TNF-α is responsible for maintaining chemotaxis in fibroblasts and inflammatory cells. ^11^ Inflammatory cytokines, such as TNF-α and interleukins, play a crucial role in the first 48 h of normal pulp tissue healing. They not only attract inflammatory cells and stem/progenitor cells but also trigger a series of events that lead to tissue regeneration, reparative dentin formation, or both. ^12^

Traditionally, calcium hydroxide has been the preferred material for vital pulp therapy. At pH 12, calcium hydroxide (Ca(OH) _2_) is an alkaline substance that has been shown to have bactericidal effects and the capacity to stimulate the growth of hard tissue in human teeth. Ca(OH) _2_ is widely used in vital pulp treatment. Nevertheless, Ca(OH) _2_ has a number of drawbacks, such as superficial necrosis of the pulp and surrounding tissues, poor sealing and adherence to dentin, unpredictable dentinal bridge formation, and tunnel defects in these bridges that could serve as possible entry points for bacteria. Because of this weakness, scientists are searching for substitute materials with antimicrobial properties that can be mixed with calcium hydroxide. ^13^^,^^14^

In the past two years, Tetraethyl Orthosilicate (TEOS), Carbonated Hydroxyapatite (CHA), chitosan (CH), and gelatin (G) have been used to create composite materials. A silane-based crosslinker that can establish bonds with both inorganic and organic materials is called TEOS. The inorganic mineral Ca(OH) _2_ found in this study’s composite material, gelatin, chitosan, TEOS, and Ca(OH) _2_, bonds to polymer chains in a naturally occurring organic polymer matrix (gelatin and chitosan) by the action of TEOS, a crosslinker. Silicon (Si) found in TEOS binds as siloxane to form a silica core that can prevent the spontaneous release of Ca(OH) _2_. This substance can be used for medication delivery with good stability. ^15^

This study aimed to determine the effect of composite materials with gelatin (G), chitosan (CH), Tetraethyl Orthosilicate (TEOS), and Ca(OH) _2_ on inflamed dental pulp. The research was done outside on mice. Evaluation was carried out on the expression of inflammatory factors, such as COX-2 expression and number of neutrophils, pulp vitality (PGP 9.5 expression), and pulp repair process (TNF-α expression).

## Methods

The Ethics Commission of the Faculty of Dentistry at Universitas Gadjah Mada approved the study procedure through the issuance of ethical clearance number No. 58/UN1/KEP/FKG-RSGM/EC/2023 on May 9, 2023. The Integrated Research and Testing Laboratory (LPPT) Unit IV, Universitas Gadjah Mada, provided experimental animals. At the Integrated Research Laboratory, Faculty of Dentistry, Universitas Gadjah Mada, the gelatin-chitosan-TEOS-Ca (OH) _2_ composite material was prepared in accordance with our earlier study. ^16^

**Figure 1.**
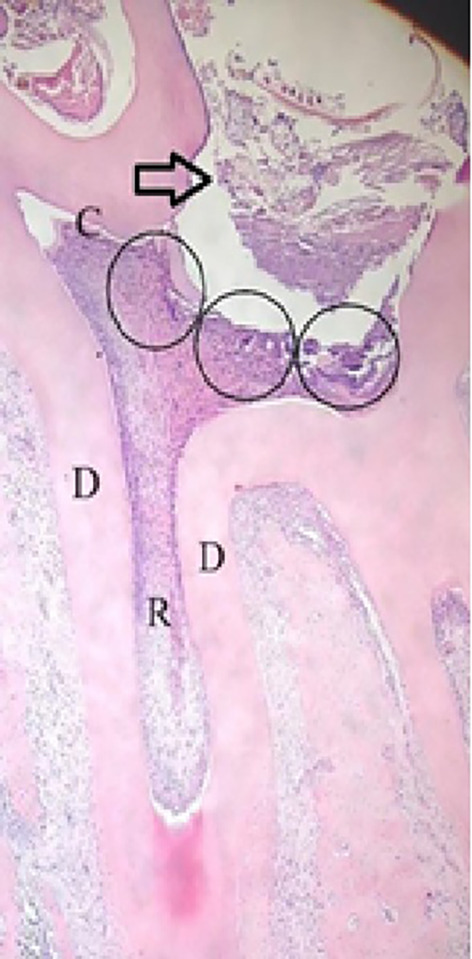
The area under the cavity preparation was observed in three fields (circle = 
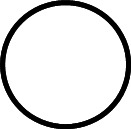
); 
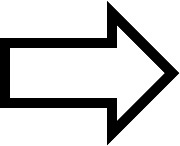
 = injury site; C = crown; D = dentin; R = root. HE Staining (Magnification ×40).

Sixteen male Wistar rats (Extended data) were used in the experiment and were divided into two groups. Eight rats in each group were divided into four groups on the first and third observation days following treatment. Before treatment and termination to Wistar rats, all rats were anesthetized using Ketamine HCl (0.1 ml/100 g body weight) (Ketamil, CV. Karebet Karya Persada, Indonesia, catalog No. 2960000999-2HH-100335784) to reduce animal suffering. ^38^ Ketamine HCl (0.1 ml/100 g body weight) was used intramuscularly to anesthetize the experimental animals, and cavities were then prepared. The maxillary first molars were prepared on the occlusal surface to pulp depth using a diamond round bur No. 010 driven by a micromotor that rotated at 35,000 rpm. The treatment group was treated with Gelatin, Chitosan, TEOS, and Ca(OH) _2_ composite, ^37^^,^^38^ whereas the control group was treated with Ca(OH) _2_ (Brazilian Biodinamica brand powder). The application was performed on the base of the cavity before temporary filling was used to fill it ( Figure 1). ^36^

### Histological preparation

The rats were administered ketamine HCl (0.1) and xylene ml/100-gram body weight) intramuscularly on the thigh to induce anesthesia on the first and third days following treatment. The jaws were fixed for four weeks at 4°C in 10% buffered formalin and decalcified with 10% EDTA (pH 7.4) (Extended data). The next step involved tissue cutting, histological preparation, and the creation of paraffin blocks. Using a microtome (Accu-Cut ^®^ SRM ^TM^ Rotary Microtome, Sakura Finetechnical Co., Ltd, Japan), paraffin blocks were sliced into 4 μm thick pieces for hematoxylin-eosin and immunohistochemistry (IHC) staining.

### Immunohistochemical staining

The paraffin sections were dewaxed three times for five minutes apiece using xylene, and the rehydration process was then carried out using 100% alcohol for five minutes, and 95%, 90%, 80%, and 70% for three minutes each. An immunohistochemistry kit (Elabscience, USA) was used for IHC staining. To remove endogenous peroxidase activity, samples were incubated with 3% H _2_O _2_ for 10 min. The samples were then washed thrice in PBS for two minutes. After adding Normal Goat Blocking Buffer (Elabscience, USA), the samples were incubated for 30 min at 37°C (Extended data). The samples were shaken to remove excess liquid. Primary antibodies against COX-2 (Rabbit Polyclonal antibody COX-2, NB100-689, Novusbio, USA), TNF-α (Rabbit Polyclonal Antibody TNF-α, bs2081R, Bioss Antibody, USA), and PGP 9.5, (Monoclonal Mouse Antibody PGP 9.5, MAB60072, R&D Systems, USA) were added at a proper dilution ratio of 1:100 in PBS. Overnight Incubation was performed at 4°C. The next day, the cells were washed three times with PBS washes for two minutes. Polyperoxidase-anti-Mouse/rabbit IgG (Elabscience, USA) was added to the samples, which were then incubated for 20 min at room temperature. PBS was used for washing three times for two minutes. After adding 3,3′-diaminobenzidine (DAB) to the samples, the DAB coloring process was closely monitored until the color of the samples turned brown, indicating a positive result. The chromogenic reaction was stopped by washing the sections in deionized water, after which hematoxylin was used for counterstaining.

### Observation

Using a light microscope at 200× magnification, COX-2 expression, PGP 9.5, and TNF-α were identified as varying degrees of brown staining, primarily found in the cytoplasm and nuclear membrane of the positive cells. Three fields were observed in the prepared cavities ( Figure 1). ^36^ ImageJ software (National Institutes of Health) free access, version 154, was used for the image analysis of COX-2, PGP 9.5 and TNF-α expression. Method of Counting: Three distinct ocular fields were used to count neutrophil cells in the cavity preparation area from HE staining. Expression of COX-2, PGP 9.5 and TNF-α were performed using ImageJ software on a Windows-based platform, with the aid of an additional plug-in available for free download from https://sourceforge.net/projects/ihcprofiler/. Data were analyzed using post hoc LSD and ANOVA (IBM SPSS Statistics, USA).

## Results

**Figure 2.**
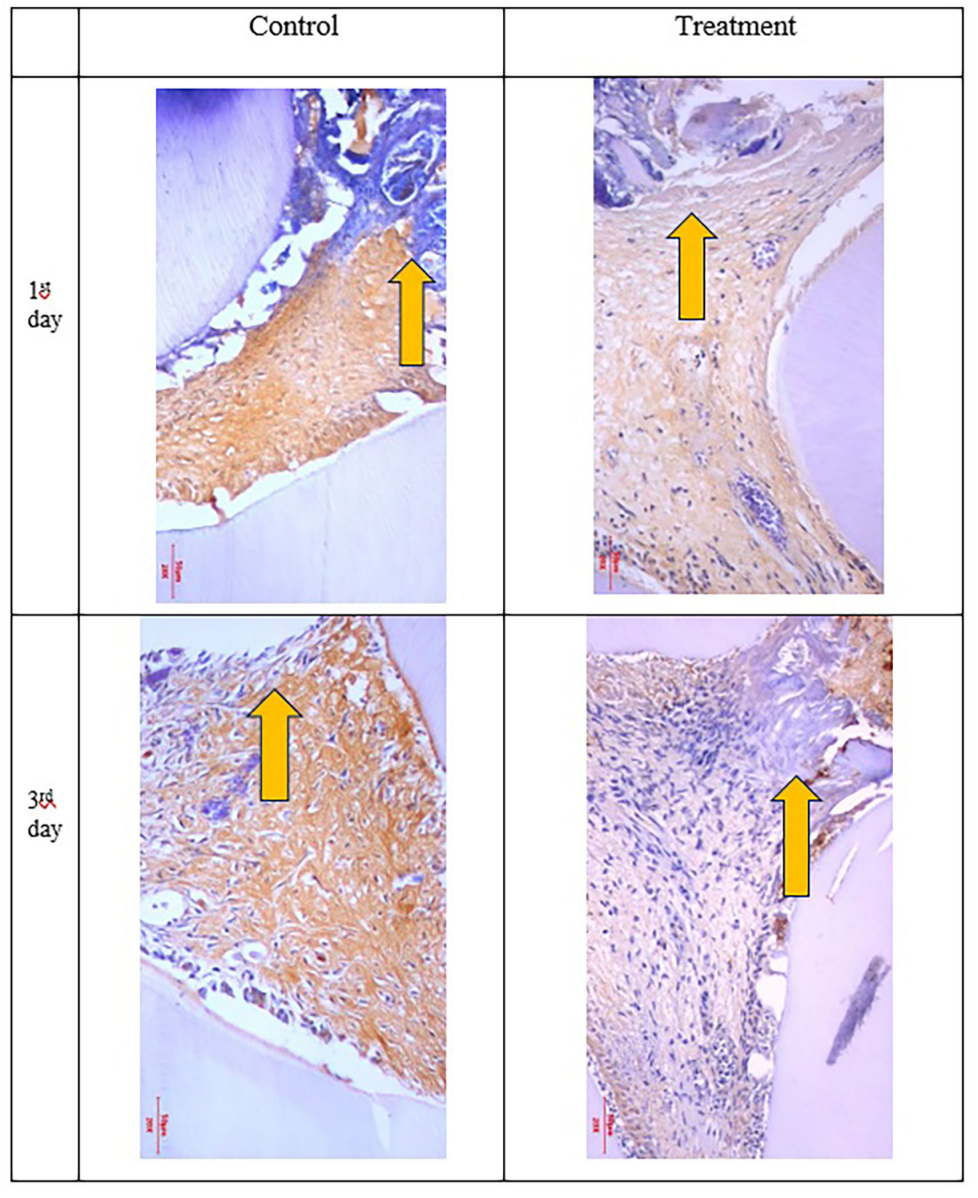
COX-2 expression appeared brown in the pulp and was weaker after administration of Gelatin-Chitosan-TEOS-Ca (OH) _2_ composite (treatment) compared to the control. D = dentin, PD = Predentin, 
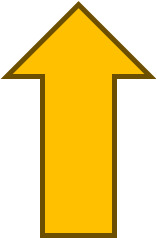
 = area of injury.

The results showed that COX-2 expression on all observation days 1 ^st^ and 3 ^rd^ was lower in the treatment group than in the control group ( Table 1). The results of immunohistochemical staining using anti-COX-2 antibodies showed a brown color in the extracellular matrix of the dental pulp with varying intensities, indicating positive COX-2 expression. The stronger the color intensity, the stronger is the COX-2 expression. Histological observations ( Figures 2 and 6) ^36^ on the 1 ^st^ day of both groups showed that there was COX-2 expression by the extracellular matrix in the area under the injury. The treatment group showed lighter color intensity than the control group. On the 3 ^rd^ day, there was a significant decrease in COX-2 expression ( Table 1) in both groups, with the color intensity in the control group being stronger than that in the treatment group ( Figure 2). On the observation day, there was no significant difference in COX-2 expression between the control and treatment groups ( Table 1). ^36^

**Table 1.** Post hoc LSD analysis of COX-2, PGP 9.5 and TNF-α after application Gelatin-Chitosan-TEOS-Ca (OH) _2_ composite (treatment) and Ca (OH) _2_ as control on the 1 ^st^ and 3 ^rd^ day.

	COX-2	PGP 9.5	TNF-α	Number of neutrophils
1 ^st^ day control - 1 ^st^ day treatment	0.099	0.001 *	0.000 *	0.414
1 ^st^ day control - 3 ^rd^ control	0.001 *	0.040 *	0.725	0.000 *
1 ^st^ day treatment - 3 ^rd^ day treatment	0.001 *	0.000 *	0.074	0.000 *
3 ^rd^ day control - 3 ^rd^ day treatment	0.076	0.000 *	0.000 *	0.430

* Significance (< 0.05).

**Figure 3.**
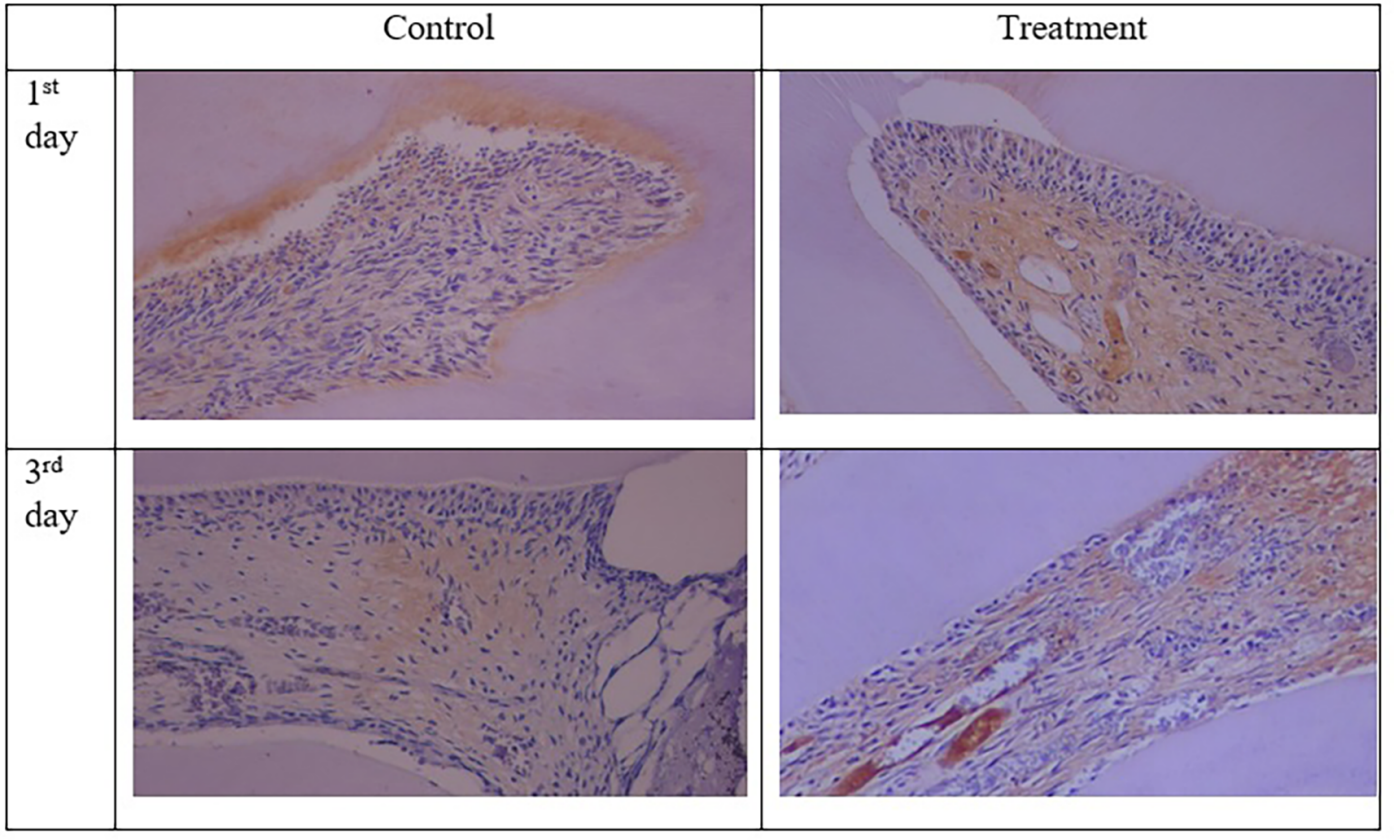
PGP 9.5 expression showed brown in the nerve pulp. Expression PGP 9.5 was stronger after administration of Gelatin-Chitosan-TEOS-Ca (OH) _2_ composite (treatment) compare to control. (Magnification ×200).

**Figure 4.**
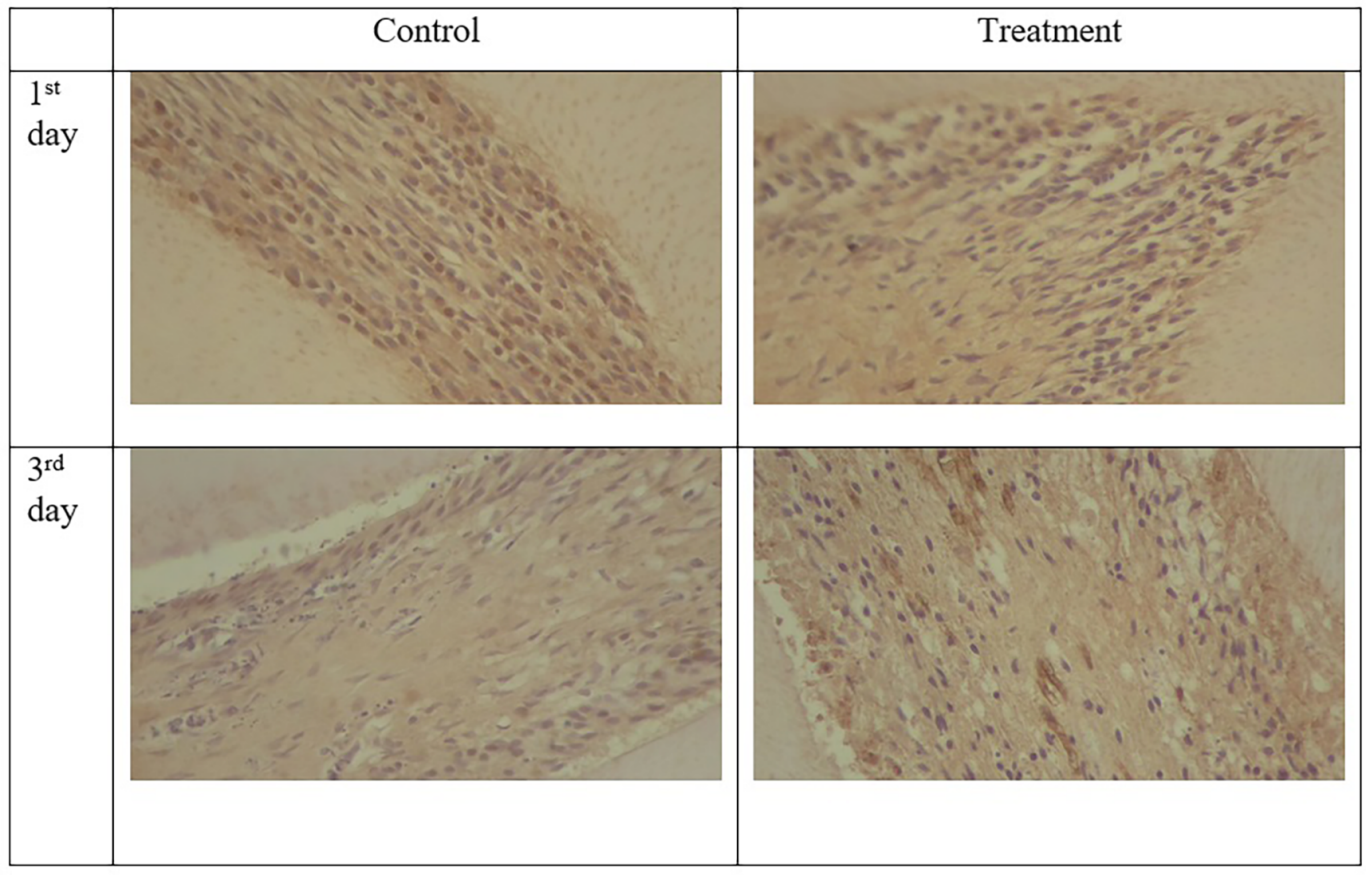
Expression of TNF-α appeared brown in the odontoblast. Positive expression was shown after administration of Gelatin-Chitosan-TEOS-Ca (OH) _2_ composite (treatment) and controls. (Magnification x200).

**Figure 5.**
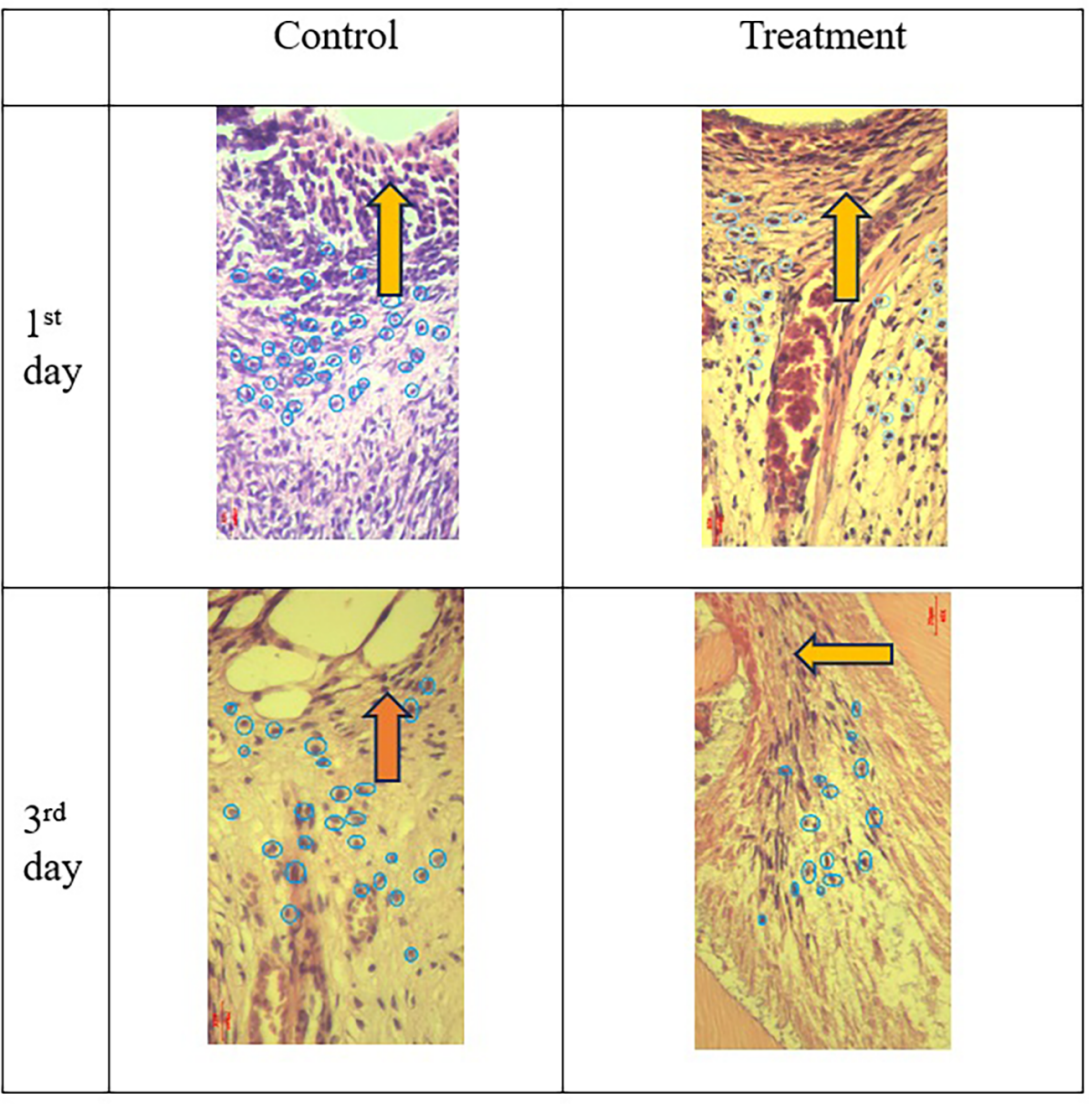
Neutrophil number (circle) in the pulp after administration of Gelatin-Chitosan-TEOS-Ca (OH) _2_ composite (treatment) were less than control (HE; 400×). Inflammation decreased with increasing observation time. 
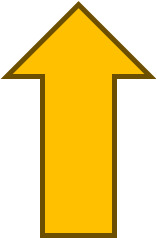
 = area of injury, 
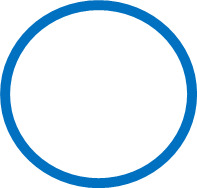
 = neutrophils.

**Figure 6.**
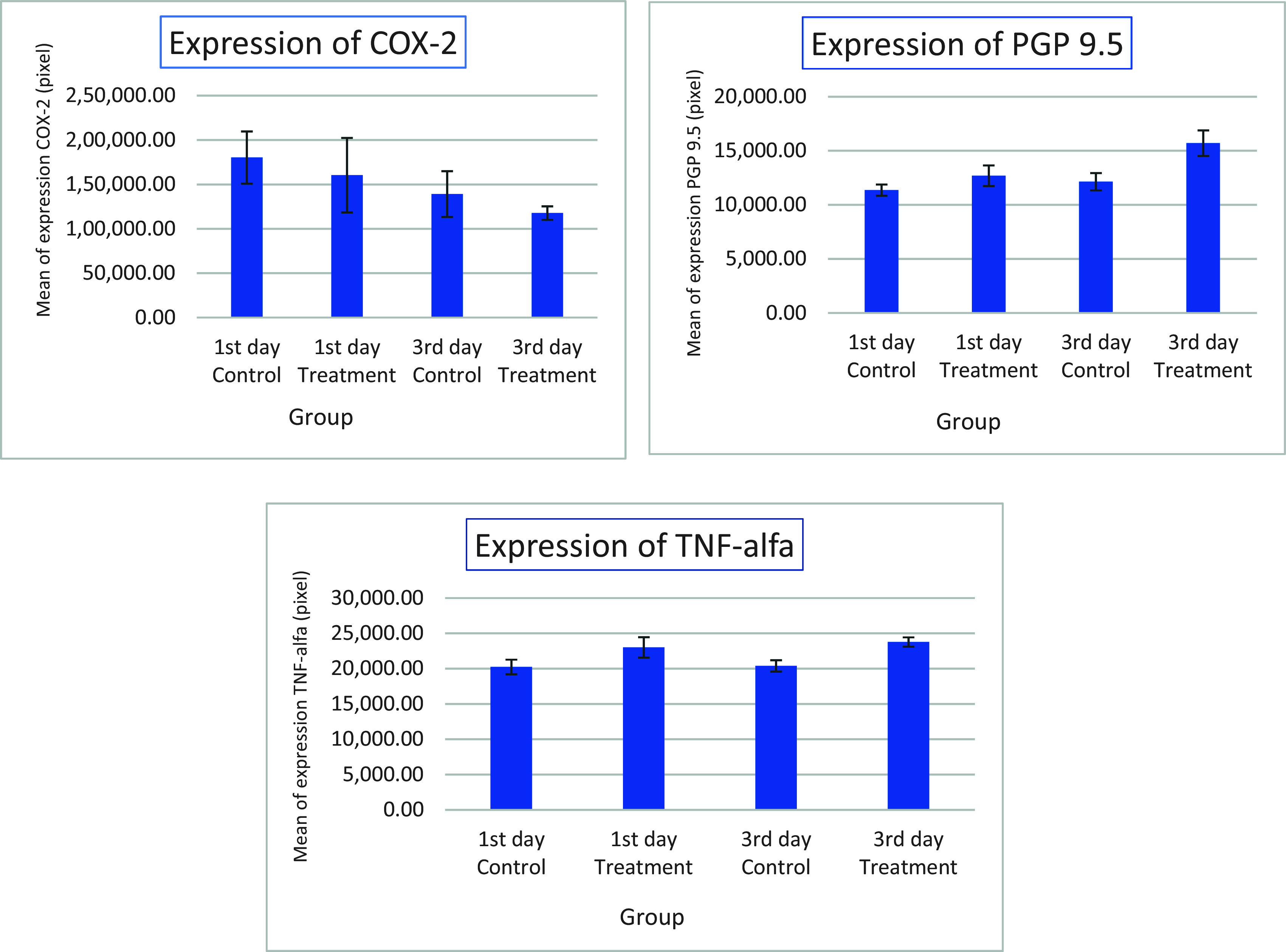
Mean and standard deviation expression of COX-2, PGP 9.5 and TNF-α after application Gelatin-Chitosan-TEOS-Ca (OH) _2_ composite (treatment) and Ca(OH) _2_ as control on the 1 ^st^ and 3 ^rd^ day.

The results for PGP 9.5 showed brown nerve pulp after administration of gelatin-chitosan-TEOS-Ca (OH) _2_ composite compared to controls ( Figure 3), ^36^ and the expression in the treatment group was significantly higher compared to controls on both observation days 1 ^st^ and 3 ^rd^ ( Table 1). The highest expression of PGP 9.5 was observed in the treatment group on 3 ^rd^ day of treatment ( Table 1).

TNF-α expression appeared brown in the odontoblasts after administration of the gelatin-chitosan-TEOS-Ca (OH) _2_ composite and controls on the 1 ^st^ and 3 ^rd^ days ( Figure 4). ^36^ The expression of TNF-α in the control group was weaker than that in the control group on 3 ^rd^ ( Figure 6). On the 1 ^st^ and 3 ^rd^ days of observation, there was no significant difference between the control group; however, TNF-α expression increased significantly between the control and treatment groups based on the day of observation ( Table 1). The highest TNF-α expression appeared on the 3 ^rd^ day of treatment ( Figure 6). ^36^

**Figure 7.**
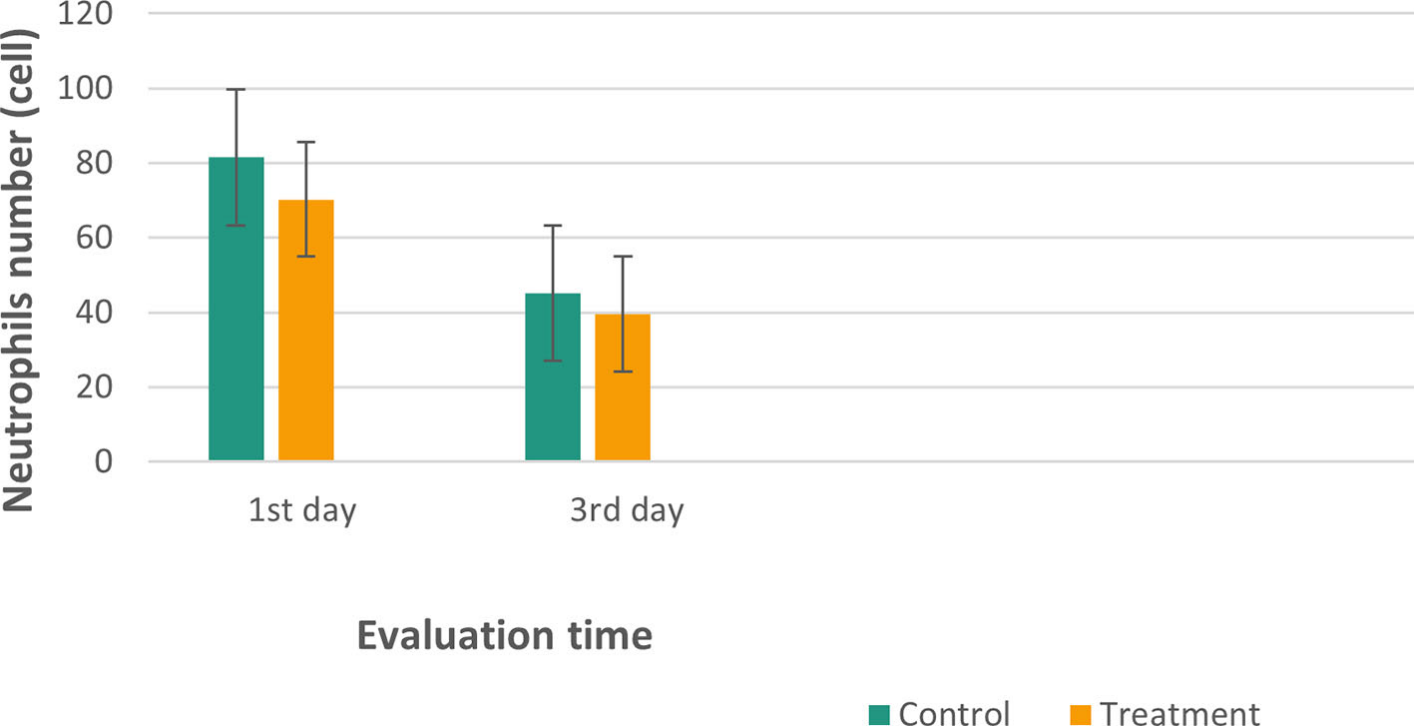
Mean and standard deviation of the neutrophils number in the inflamed pulp of *Wistar* rats.

The results of histological observations ( Figures 5 and 7) ^36^ showed that on 1 ^st^ day in the group administered Ca(OH) _2_ (control), the number of neutrophils was higher than that in the treatment group. Results of the mean neutrophil number calculation. The mean number of neutrophils in both the groups decreased significantly on the 3 ^rd^ days of observation ( Table 1).

## Discussion

The results of this study showed that the use of a composite gelatin-chitosan-TEOS-Ca (OH) _2_ medicament as pulp capping leads to increased COX-2 expression on the 1 ^st^ day then decreases significantly on the 3 ^rd^ day ( Figures 2 and 6). ^36^ These results were in line with the number of neutrophils which decreased in 3 ^rd^ day of observation ( Figure 5). These findings suggest that the gelatin-chitosan-TEOS-Ca (OH) _2_ composite material induces a lower inflammatory response.

In this study, the cavity was prepared using a low-speed rotary instrument. Every rotary cutting instrument generates heat and vibrations that may damage the tooth pulp. If heat is delivered to the pulp, it may cause histopathological alterations can occur. Excessive heat transmission may also cause pulp inflammation and irritation. ^17^^,^^18^

Inflammation of the dental pulp is a complicated process that is controlled by several molecular mediators, and the mechanism is the same as that of inflammation in other connective tissues. The primary role of these inflammatory mediators is to shield tissues from external irritants such as chemicals, mechanical forces, and germs. ^19– 21^ Injuries that result from the preparation of deep cavities can trigger the production of TNF-alpha and IL-1 by tissue-resident macrophages and fibroblasts, which can increase COX-2 expression in the injured area. ^22^

The findings demonstrated that COX-2 expression was discernible in the extracellular matrix, neutrophils, macrophages, lymphocytes, and fibroblasts in the region of injury. Tissue damage, such as that caused by surgery or conditions such as pulpitis or periodontitis, causes the creation of COX-2, which in turn causes the synthesis of prostaglandins that sensitize pain fibers and encourage inflammation. ^23^ PGE2 and prostacyclin are products of COX-2 activity and are implicated in various physiological and pathological processes, including inflammation and pain. ^24^

The dental pulp can be impacted and harmed by exogenous irritants, such as mechanical, chemical, and bacterial agents, because they can activate the pulp’s innate and adaptive immune responses in addition to a variety of molecular mediators. Damage resulting from prepared deep holes has the potential to activate tissue-resident macrophages and fibroblasts, causing them to release TNF-α and IL-1, which in turn can elevate COX-2 expression in injured areas. ^25^

There was a significant difference between the 1 ^st^ and 3 ^rd^ day of treatment as well as between the 1 ^st^ and 3 ^rd^ day of control, and the COX-2 color intensity in the treatment groups was lower on the first and third days compared to the control group. According to Jiang et al., ^26^ this outcome is assumed to be caused by the antibacterial and anti-inflammatory qualities of chitosan, which can reduce the bacterial load and reduce the initial inflammatory insult. The regulated release of Ca(OH) _2_ is facilitated by the matrix offered by gelatin. This minimizes the risk of excessive initial burst release, limits the entry of pathogens, and reduces the requirement for acute inflammatory cell infiltration. Both gelatin and Ca(OH) _2_ exhibit anti-inflammatory properties. ^27, 28^ The composite gelatin-chitosan-TEOS-Ca (OH) _2_ provides an environment that limits post-treatment inflammation in the pulp tissue, as seen by the lowered levels of these proinflammatory cytokines and mediators. (Extendd data).

The evaluation conducted on the first day post-treatment aided in determining the immediate reaction of COX-2 expression to the therapy, and we observed a non-significant decrease in COX-2 expression in the treatment group when compared to the control. The evaluation on the third day aided in tracking any short-term variations and possible trends in COX-2 expression after the first reaction, where the mean expression was much lower than on the first day.

After taking these factors into consideration, the composite gelatin-chitosan-TEOS-Ca (OH) _2_ material seems to help the pulp by reducing the initial inflammatory insult and, due to the synergistic effects of its bioactive components, fostering a more favorable healing environment. As the number of observation days increased, we saw a drop in the pattern of COX-2 expression. The overall pattern was reduced on the third day when compared to the first, with a significant decrease between the control and treatment groups ( Table 1). On the 3 ^rd^ day, a comparison of COX-2 expression and neutrophil counts between the treatment and control groups revealed no discernible differences. This suggests that the reduction in acute inflammation caused by the gelatin-chitosan-TEOS-Ca (OH) _2_ material is equivalent to that of Ca(OH) _2_. It is crucial to remember that further investigation and assessment are required to validate the long-term results and clinical effectiveness of this composite material. Specifically, the ratios of gelatin and chitosan within the material need to be changed to enhance their anti-inflammatory properties and maximize their efficacy.

The presence of chitosan may be the reason for the notable increase in PGP 9.5 expression on the first and third days after injection, which is consistent with other research showing the regeneration potential of chitosan-based composites. Chitosan, a natural copolymer of glucosamine and N-acetylglucosamine, is used in gene therapy, tissue engineering (TE), and other biomedical fields because of its biocompatibility, stability, sterilizability, biodegradability, antimicrobial activity, and immunostimulatory activity. ^29^

Chitosan also offers a biomimetic microenvironment that promotes cell growth owing to its similarity to glycosaminoglycans (GAGs) found in naturally occurring extracellular matrix (ECM) materials. Furthermore, its osteoconductive properties can support osteogenic differentiation and biomineralization of stem/progenitor cells. ^29^ The shortcomings of chitosan in terms of mechanical strength and early cell attachment can be effectively addressed by blending it with other biomaterials, such as gelatin. ^29^ A protein fragment known as gelatin is produced when collagen fibers partially break down. Its numerous benefits, such as low antigenicity, biodegradability, hydrogel qualities, and affordability, have made it widely available in TE. Additionally, the Arg–Gly–Asp (RGD) integrin recognition motif supports the first cell attachment. Blends of chitosan and gelatin, or CS/Gel, have been suggested as scaffolding materials for bone regeneration and other tissues, including skin, cartilage, and peripheral nerves. ^29^ These blends can potentially combine these features.

The incorporation of calcium hydroxide (Ca(OH) _2_) and tetraethyl orthosilicate (TEOS) into the composite may increase its effectiveness by promoting cellular adhesion, proliferation, and extracellular matrix formation. It is probable that the composite material triggered an early neurogenic response by triggering signalling pathways linked to cell differentiation and proliferation. Therefore, the persistent increase in PGP 9.5 expression may be a sign of continued axonal development and growth, which would ultimately aid in functional recovery. ^29^ The study findings confirmed that the treatment group receiving gelatin, chitosan, TEOS, and Ca(OH) _2_ had the highest levels of PGP 9.5 expression.

Two distinct receptors attach to TNF-α and initiate signal transduction pathways. These pathways trigger numerous biological responses such as cell survival, differentiation, and proliferation. ^30^ After administering the gelatin-chitosan-TEOS-Ca (OH) _2_ composite and Ca(OH) _2_ to the control group, odontoblasts were shown to express TNF-α, indicating that both materials triggered an inflammatory response. The brown coloration observed indicates an upregulation of TNF-α production, which is a key proinflammatory cytokine involved in various cellular processes, including tissue repair and inflammation. On the 1 ^st^ and 3 ^rd^ days, there was a similar expression of TNF-α in the treatment and control groups, indicating that the level of inflammatory response caused by both materials in tooth pulp was equivalent. This result is consistent with other research showing that Ca(OH) _2_ has strong immunomodulatory effects in dental pulp tissues, which upregulates TNF-α expression. ^30^

On the third day, the control group showed lower TNF-α expression than the treatment group, which was remarkable. This disparity could point to variations in the TNF-α expression kinetics between the gelatin-chitosan-TEOS-Ca (OH) _2_ composite and pure Ca(OH) _2_. Compared with pure Ca(OH) _2_, the composite material might have produced a stronger inflammatory response or maintained TNF-α expression for a longer period of time. Further research is necessary to clarify the underlying mechanisms causing this variation in TNF-α expression between the two groups.

On the 1 ^st^ day, infiltration of inflammatory cells, especially neutrophils, was observed in both groups. Neutrophils are leukocytes that first migrate to tissue/injury locations. These cells are dominant within 24-36 hours after injury and function to eliminate irritants and damaged tissue through phagocytosis. ^31, 32^ Changes in the endothelium surface caused by the activation of inflammatory mediators, such as histamine, cysteinyl-leukotrienes, and cytokines generated by tissue-resident leukocytes during damage can trigger the recruitment of neutrophils to the injured area. ^31^ On the first and third days, the mean neutrophil count of the treatment group was lower than that of the control group. These results are in line with earlier research showing the inherent anti-inflammatory properties of the constituent parts of our composite, such as chitosan, ^33^ gelatin, ^28^ and Ca(OH) _2_. ^29^ Post-treatment inflammation of the pulp tissue was inhibited by the composite gelatin-chitosan-TEOS-Ca (OH) _2_, as evidenced by the decreased levels of proinflammatory cytokines and mediators. This suggests that pulp-capping methods result in better pulp healing and regeneration rather than necrosis or infection, which could compromise the effectiveness of vital pulp therapies. ^34^ Reductions in these indicators have been linked to improved clinical results in comparable studies. This suggests that pulp-capping treatments result in better pulp healing and regeneration rather than necrosis or infection, which compromises the effectiveness of vital pulp therapy procedures. ^35^

The gelatin-chitosan-TEOS-Ca (OH) _2_ composite may facilitate a rapid resolution of inflammation and stabilization of the pulp capping wound site by restricting early neutrophil infiltration. Based on the results obtained, we conclude that the gelatin-chitosan-TEOS-Ca (OH) _2_ composite and Ca(OH) _2_ evoke similar biological responses in terms of neutrophil number, with no significant difference observed between the treatment group and the control group every day of observation. The results of the study also showed that gelatin-chitosan-TEOS-Ca(OH) _2_ composite can stimulate the pulp repair process and pulp vitality as indicated by the expression of TNF-alpha and PGP 9.5. TNF-α expression as a response to inflammation may also affect the formation of dentinal bridges by modulating cellular responses and extracellular matrices.

The results of this study support previous studies that the use of gelatin-chitosan-TEOS-Ca(OH) _2_ composite has the potential as a medicament for exposed pulp. This is evidenced by its ability to carry Ca(OH) _2_ more effectively by reducing the alkaline properties indicated by weak positive Nestin expression around the irritated pulp. This indicates the proliferation and differentiation of odontoblast cells and cells in the pulp towards tissue regeneration. ^16^ To completely understand the therapeutic effectiveness and underlying mechanisms action of gelatin-chitosan-TEOS-Ca (OH) _2_ composite in endodontic applications, more research is required including its effects on odontoblast-like cells and TGF beta.

## Conclusion

The gelatin-chitosan-TEOS-Ca (OH) _2_ composite material has potential as an anti-inflammatory agent in the exposed pulp by reducing COX-2 expression and the number of neutrophils. These findings indicate a possible regenerative effect of the gelatin-chitosan-TEOS-Ca (OH) _2_ composite by increasing TNF-α expression and PGP 9.5, making it a promising candidate for further exploration in dental pulp therapy.

### Ethics and consent

The Ethics Commission of the Faculty of Dentistry at Universitas Gadjah Mada approved the study procedure through the issuance of ethical clearance number No. 58/UN1/KEP/FKG-RSGM/EC/2023 on May 9, 2023.

## Data availability

### Underlying data

Figshare: Data final assignment recognition program (RTA)-2024. https://doi.org/10.6084/m9.figshare.26819887 ^36^

This project contains the following:

• Raw data
• Figure files
• Table

The data are available under the terms of the Creative Commons Attribution 4.0 International license (CC-BY 4.0).

### Extended data

Figshare: Data final assignment recognition program (RTA)-2024. https://doi.org/10.6084/m9.figshare.27021160.v1 ^37^

This project contains the following:

• Figure files

The data are available under the terms of the Creative Commons Attribution 4.0 International license (CC-BY 4.0).

### Reporting guidelines

Figshare: Data final assignment recognition program (RTA)-2024. https://doi.org/10.6084/m9.figshare.27020713.v2 ^38^

This project contains the following:

• Arrive checklist

The data are available under the terms of the Creative Commons Attribution 4.0 International license (CC-BY 4.0).
