# Supplementary material for: Evaluating the Efficacy of Gelatin-Chitosan-Tetraethyl Orthosilicate Calcium Hydroxide Composite as a Dental Pulp Medicament on  COX-2, PGP 9.5, TNF-α Expression and Neutrophil number
Source: F1000Res. 2025 Jan 30;13:1258. Originally published 2024 Oct 21. [Version 2] doi: 10.12688/f1000research.156336.2 (PMC11862375; doi:10.12688/f1000research.156336.2)
Supplement: Peer review history

# Reviewer report: Reviewer response for version 2

**DOI:** 10.5256/f1000research.177286.r363804

**Published:** 2025-02-08

**Authors:** Caroline Anselmi

**Affiliations:** 1 São Paulo State University, Araraquara, Brazil; 2 Department of Cariology, Restorative Sciences & Endodontics, University of Michigan School of Dentistry, Ann Arbor, Michigan, USA

This study explores a novel pulp capping material composed of gelatin (G), chitosan (CH), tetraethyl orthosilicate (TEOS), and calcium hydroxide [Ca(OH) _2_], collectively referred to as G-CH-TEOS-Ca(OH) _2_. The authors evaluated the effect of this novel material in pulp capping of Wistar rats compared to the gold standard (Ca(OH) _2_). Given the clinical importance of vital pulp therapy and the ongoing search for improved alternatives to Ca(OH)2 in exposed pulp treatments, this material holds potential for advancing therapeutic outcomes. However, the paper lacks clarity, and the data presentation is flawed. Key areas requiring improvement are outlined below.

  

1. Abstract: Results: The authors mention, “The results showed a decrease in COX-2 expression, but not significantly”. If there was no significant difference between the treatment and the control group, your results did not show a decrease in COX-2. The same applies to neutrophil numbers.
2. More information about the G-CH-TEOS-Ca(OH) _2 _composition, characterization, and mechanism of action should be mentioned in the introduction.
3. Table 1 is confusing; the information about the differences found in the post hoc should be presented in the graphs using different letters or symbols. The p-value should be presented in the results section.
4. Figures 6 and 7 are missing statistical analysis in the graph and the figure caption
5. Data presented in figures 6 and 7 should be analyzed using Two-way ANOVA since you presented 2 factors (time point and treatment).
6. The conclusion does not represent the results found in the paper since there were no differences in the COX-2 expression and number of neutrophils when compared to the control group.

Is the work clearly and accurately presented and does it cite the current literature?

Partly

If applicable, is the statistical analysis and its interpretation appropriate?

No

Are all the source data underlying the results available to ensure full reproducibility?

Partly

Is the study design appropriate and is the work technically sound?

Yes

Are the conclusions drawn adequately supported by the results?

No

Are sufficient details of methods and analysis provided to allow replication by others?

Yes

Reviewer Expertise:

Pulp biology, in vitro and in vivo analysis of biomaterials, cytotoxicity of dental materials.

I confirm that I have read this submission and believe that I have an appropriate level of expertise to confirm that it is of an acceptable scientific standard, however I have significant reservations, as outlined above.

# Response

**Published:** 2025-02-12

**Authors:** Juni Handajani

**Affiliations:** Universitas Gadjah Mada, Indonesia

Dear reviewer,

 Thank you very much for your valuable suggestions.

 We revised the manuscript according to the reviewer's suggestion in the Abstract Conclusion by removing the number of neutrophils.

 In the introduction, an explanation has been added regarding the composition of the G-CH-TEOS-Ca (OH)2 material in accordance with previous studies.

 The results of the study have been added to the explanation of the statistical analysis of homogeneity, normality and ANOVA analysis.

 Changes are also made to the contents of Table 1 which previously contained the results of the Post hoc LSD analysis to the results of the ANOVA analysis, while the results of the Post hoc LSD analysis are shown in Figure 6.

 Figure 7 which previously displayed the graph of the number of neutrophils has been removed. The graph of the number of neutrophils has been moved and combined with Figure 6.

# Reviewer report: Reviewer response for version 1

**DOI:** 10.5256/f1000research.171629.r354056

**Published:** 2025-01-16

**Authors:** Euis Reni Yuslianti

**Affiliations:** 1 Biology Oral and Biomedicine Department, Universitas Jenderal Achmad Yani, Cimahi, West Java, Indonesia

The manuscript is clear and logically organized, but please more recent references and a deeper discussion of **alternative pulp-capping materials.**

 The methodology is mostly sufficient, please more **details on composite preparation and ethical handling of animals**.

 The discussion could benefit from clearer emphasis on **the clinical relevance of the findings**. Please explicitly stating **how the composite material could be superior to traditional Ca(OH)2-based treatments in vital pulp therapy.**

 The explanation of TNF-α expression kinetics, could include additional references to ensure claims are fully substantiated.

 Please deep discussion how the **observed inflammatory responses might affect long-term outcomes **(e.g., pulp vitality, dentin bridge formation).

Is the work clearly and accurately presented and does it cite the current literature?

Yes

If applicable, is the statistical analysis and its interpretation appropriate?

Yes

Are all the source data underlying the results available to ensure full reproducibility?

Yes

Is the study design appropriate and is the work technically sound?

Yes

Are the conclusions drawn adequately supported by the results?

Yes

Are sufficient details of methods and analysis provided to allow replication by others?

Yes

Reviewer Expertise:

Oral biology, biomedical, oral biochemistry, herbal medicine in dentistry

I confirm that I have read this submission and believe that I have an appropriate level of expertise to confirm that it is of an acceptable scientific standard.

# Response

**Published:** 2025-01-21

**Authors:** Juni Handajani

**Affiliations:** Universitas Gadjah Mada, Indonesia

Dear Reviewer,

 Thank you for your valuable sugggestion to improve our manuscript.

 We have revised the discussion by adding previous research findings that support the results of this study.

 Thank you.

# Reviewer report: Reviewer response for version 1

**DOI:** 10.5256/f1000research.171629.r341881

**Published:** 2024-12-19

**Authors:** Angela Quispe-Salcedo

**Affiliations:** 1 Niigata University, Niigata, Japan

The authors of this study aimed to evaluate the effect of a calcium hydroxide gelatin-chitosan-tetraethyl orthosilicate composite material on pulp inflammation in rats.

 This is a descriptive morphological study, in which results were collected on days 1 and 3 after surgery. Although this is an interesting idea, the usefulness of this material needs further research and analysis. This manuscript contains several errors that the author needs to improve before considering for indexing.

 1. Title: Please remove the parentheses and write a brief title that summarizes the purpose of your research

 2. The introduction is too long and the paragraphs are too short.

 Please pay attention to the content and combine paragraphs 1 and 2, and 6 and 8.

 The information in paragraph 7 should be linked back to paragraphs 1 and 2, for example ,

 In paragraph 9, I suggest adding the information that the research was done outside on mice (line 2 of paragraph 9).

 3. Method: Why did the author use a micromotor instead of a handpiece under refrigeration?

 In this way, pulp damage is greater, is this done intentionally under high temperature conditions?

 Why did the author choose days 1 and 3?

 The initial repair phase, takes place from day 5.

 Days 1 and 3 are for observation of inflammatory reactions.

 There is no morphological assessment of pulp tissue. H-E staining is required.

 Did the author mean Xylene instead of Xylol?

 Please explain.

 The authors did not adequately explain how they quantified the IHC results and how they performed the neutrophil quantification.

 The description of “yellowish to brownish color grades” is unacceptable.

 Brand and company name information is missing from the descriptions of some reagents.

 Information about statistical software is also missing.

 4. Results:

 The results of this study were not convincing, nor were they properly presented in the manuscript. To name just a few of the main concerns, what the authors claimed were positive antibody responses, in fact appeared to be nonspecific responses distributed throughout the pulp tissue. The images were not standardized, and it was difficult to observe the immune response at this magnification.

 In Fig. 3, PGP-9.5 should be located in the nerve fibers of the pulp tissue, but there is no evidence of nerve fibers. Figure 4 is very poor, the image is very dark and there is nothing to compare.

 Figure 5 is related to the presence of neutrophils but we cannot assess them at this magnification and the samples show different locations so the images cannot be compared or read.

 The graph in Figure 6 must be split into three independent graphs for each marker and finally linked to Figure 7, there is no measurement or unit of analysis for the number of neutrophils on the Y axis. We, the readers, do not understand how the author calculated it.

 5. Discussion

 Unnecessary long, the paragraphs are not connected. Please summarize and discuss in detail your results compared to previous studies.

 This section needs to be completely rewritten.

 6. Conclusion

 Unfortunately, in its current form, the results of this manuscript cannot support the conclusions drawn by the authors.

Is the work clearly and accurately presented and does it cite the current literature?

Partly

If applicable, is the statistical analysis and its interpretation appropriate?

Partly

Are all the source data underlying the results available to ensure full reproducibility?

Partly

Is the study design appropriate and is the work technically sound?

No

Are the conclusions drawn adequately supported by the results?

No

Are sufficient details of methods and analysis provided to allow replication by others?

Partly

Reviewer Expertise:

Dental pulp biology

I confirm that I have read this submission and believe that I have an appropriate level of expertise to state that I do not consider it to be of an acceptable scientific standard, for reasons outlined above.

# Response

**Published:** 2025-01-21

**Authors:** Juni Handajani

**Affiliations:** Universitas Gadjah Mada, Indonesia

Dear Reviewer,

 Thank you for the very valuable input to improve our manuscript.

 We have made revision according to the reviewer's suggestion, including:

 1. Revision of the research title

 2. Rewritten of the introduction

 3. Addition of explanations on research methods and observations

 4. Revision of research results and Figures 1, 6 and 7, and descriptions

 5. Addition of research discussion and references

 6. Revision of the conclusion

 Thank you
